# Design and Validation of Tools for Microbial Synthetic Biology Applications

**DOI:** 10.3390/life11080739

**Published:** 2021-07-24

**Authors:** Catarina C. Pacheco, Filipe Pinto

**Affiliations:** 1 i3S—Instituto de Investigação e Inovação em Saúde, Universidade do Porto, 4200-135 Porto, Portugal; 2 IBMC—Instituto de Biologia Molecular e Celular, Universidade do Porto, 4200-135 Porto, Portugal; 3 School of Biological Sciences, University of Edinburgh, Edinburgh EH9 3FF, UK; 4 Centre for Synthetic and Systems Biology, University of Edinburgh, Edinburgh EH9 3FF, UK

Synthetic Biology (SynBio) is a multidisciplinary field that brings together science, technology and engineering to expedite the design, creation and modification of genetic materials to be applied in living organisms or in vitro systems. This revolutionary research paradigm is guided by the engineering concepts of abstraction, decoupling and standardisation, aiming at the rational design of systems with predictable behaviour. However, the complexity of biological systems renders the implementation of SynBio a difficult endeavour, which relies on an iterative “Design–Build–Test–Learn” process. As a result, the attempts to create new functions or modify existing ones has led to novel findings that defy our current fundamental understanding of biological processes. The successful implementation of SynBio also extends to applied science for the production of a myriad of compounds and regulatory frameworks, with applications ranging from bioremediation, biofuels, cosmetics and food to disease diagnostics and treatment.

The chassis plays a key role in this process of engineering biological systems, working as the vessel that harbours the synthetic devices and circuits. Over the past two decades, the development of SynBio applications was mostly focused on the use of “universal” chassis, such as *Escherichia coli*, *Bacillus subtilis* and *Saccharomyces cerevisiae* (*S. cerevisiae*), that have been widely tamed and their lifestyle is well adapted to laboratorial conditions. However, it is becoming increasingly clear that “universal” chassis prototypes need to be overhauled by a repertoire of organisms that exhibit robust performances under the different conditions and requirements imposed by specific applications. The success of a chassis is inherently underpinned by the number and diversity of the customized biological parts that compose its molecular toolbox, as well as the methodologies available that enable the efficient implementation and predicted behaviour to achieve the desired outcome. In this context, this Special Issue aims at gathering knowledge on the design and validation of SynBio tools for microorganisms, with emphasis on computer-aided design of biological parts, genome-editing and performance evaluation, and on the development of cloning suites or high-throughput validation methods.

Rainha et al. [1] present an overview on polyphenols from the biological activities and biosynthesis to the heterologous production. Special focus is given to the strategies to optimize the production of these compounds in *S. cerevisiae*, including genome and enzyme engineering. Other aspects for the improvement of strains for polyphenol production are reviewed, namely the overproduction of amino acids, the use of extender substrates or the expression of transporters. An outline of the different SynBio methodologies that can be employed for improved implementation is provided in this work.

The fundamental SynBio tools available for the model chassis *S. cerevisiae* and other underused strains such as *Yarrowia lipolytica, Kluyveromyces marxianus, Issatchenkia orientalis* and *Pichia pastoris* is reviewed by Lacerda et al. [2]. The bioproduction of various chemicals, including ethanol and fatty acids using these chassis is presented, addressing the advantages of using non-conventional yeasts. Future developments and enabling technologies for the optimization of pathways and hosts are also discussed.

Sureda-Vives and Sarkisyan [3] summarise the application of bioluminescence-based technologies that are routinely used as reporter systems and, more recently, as tools for powerful in vivo imaging. The use of luciferins and luciferases as bioluminescent tools to control biological processes as well as the use luminopsins and photosensitisers delivering-light proteins in optogenetics applications is debated. The future directions of these bioluminescence-driven technologies for the control and regulation of complex intracellular and cell–cell interactions are featured.

The use of Clustered Regularly Interspaced Short Palindromic Repeats and CRISPR-associated protein 9 (CRISPR-Cas9) tools for editing the *S. cerevisiae* genome is reviewed, discussing in detail the different system components (e.g., the Cas9 protein or the guide RNA) and the available toolkits [4]. The authors also emphasize the potential and versatility of this methodology by revising different applications, from gene knock-ins and knock-outs to the development of cell factories through the implementation of heterologous pathways and tolerance engineering using the conventional yeast as a chassis.

As the SynBio field progresses, designs with increasing complexity and sophistication are being pursued and the input from in silico tools for advanced computer aided design, modelling, computer simulations and experiments is becoming crucial. In an original article, Karas and Hetch [5] describe a strategy to produce novel α-helical proteins by combining rational design and binary patterning of polar and nonpolar amino acids. This method enables the generation of DNA libraries encoding proteins with proper folding and stability, which provide novel sequences suited for screening and selection of functional activities in vitro and in vivo.

We expect that the contributions to this Special Issue will provide the reader with an updated view of some important aspects of SynBio, capturing the attention of both specialists and non-specialists who may be interested in pursuing further studies and practical applications in this exciting field, as well as of researchers with a broader interest.
